# Segmentation of the Himalayas as revealed by arc-parallel gravity anomalies

**DOI:** 10.1038/srep33866

**Published:** 2016-09-21

**Authors:** György Hetényi, Rodolphe Cattin, Théo Berthet, Nicolas Le Moigne, Jamyang Chophel, Sarah Lechmann, Paul Hammer, Dowchu Drukpa, Soma Nath Sapkota, Stéphanie Gautier, Kinzang Thinley

**Affiliations:** 1 Institute of Earth Sciences, University of Lausanne, Géopolis, Quartier UNIL-Mouline, 1015 Lausanne, Switzerland; 2 Department of Earth Sciences, ETH Zürich, Soneggstrasse 5, 8092 Zürich, Switzerland; 3 Laboratoire de Géologie, Ecole Normale Supérieure, CNRS - UMR 8538, 24 rue Lhomond, 75005 Paris, France; 4 Géosciences Montpellier, CNRS UMR5243, Université de Montpellier, Place E. Bataillon, 34095 Montpellier, France; 5 Department of Earth Sciences, Uppsala University, Villavägen 16, 75236 Uppsala, Sweden; 6 Department of Geology and Mines, Ministry of Economic Affairs, PO Box 173 Thimphu, Bhutan; 7 armasuisse, Science & Technology, Federal Department of Defence, Civil Protection and Sport, Feuerwerkerstrasse 39, 3602 Thun, Switzerland; 8 SwissRe, Mythenquai 50/60, 8022 Zürich, Switzerland; 9 Department of Mines and Geology, Lainchaur, Kathmandu, Nepal; 10 National Land Commission, Kawang Jagsa, PO Box 142 Thimphu, Bhutan

## Abstract

Lateral variations along the Himalayan arc are suggested by an increasing number of studies and carry important information about the orogen’s segmentation. Here we compile the hitherto most complete land gravity dataset in the region which enables the currently highest resolution plausible analysis. To study lateral variations in collisional structure we compute arc-parallel gravity anomalies (APaGA) by subtracting the average arc-perpendicular profile from our dataset; we compute likewise for topography (APaTA). We find no direct correlation between APaGA, APaTA and background seismicity, as suggested in oceanic subduction context. In the Himalayas APaTA mainly reflect relief and erosional effects, whereas APaGA reflect the deep structure of the orogen with clear lateral boundaries. Four segments are outlined and have disparate flexural geometry: NE India, Bhutan, Nepal & India until Dehradun, and NW India. The segment boundaries in the India plate are related to inherited structures, and the boundaries of the Shillong block are highlighted by seismic activity. We find that large earthquakes of the past millennium do not propagate across the segment boundaries defined by APaGA, therefore these seem to set limits for potential rupture of megathrust earthquakes.

Most of the largest earthquakes occur in subduction zones: on the Chile margin the 1960 Valdivia earthquake reached 9.5 on the Richter magnitude scale and more recently, in 2004 and 2011, magnitude 9 earthquakes have struck Sumatra and Japan. Major earthquakes also happen in the context of continental collision: in the Himalayas the 1950 Assam earthquake was estimated at magnitude Mw 8.6 (ref. 1). Several studies suggest that a historical event near 1100 AD could have reached magnitude 9 along the Himalayan front^2^,^3^. Such large events are also required in the Himalayas to balance the moment deficit derived from geodetic strain measurements^4^.

The three key parameters controlling the magnitude of an event are the co-seismic slip, the width and the length of the ruptured zone. While slip is mainly dictated by convergence rate and inter-seismic coupling between the two plates, rupture width and length are geometrical properties that can be assessed from the structure of the subduction or collision zone. The more the plate interface is continuous and homogeneous along strike, the easier it will be for rupture to propagate and extend laterally. Therefore, assuming uniform coupling^5^, segmentation of subduction and collision zones is primarily dependent on lateral variations in plate structure.

Although direct access to the plate interface is beyond reach, variations in its geometry can be reasonably assumed to be reflected in topography (e.g. ref. 6) and in the gravity field. The bending of the subducting plate determines the topographic signal through deformation of the upper plate and through the amount of sediments accumulating in the foreland basin and then entering the accretionary wedge. The gravity signal senses density contrasts at depth, and is therefore directly mapping the crust-mantle boundary geometry, as well as the thickness of the foreland basin sediments.

In oceanic subduction zones these two fields were analysed by Song and Simons^7^. Compared to the average topography and gravity (marine free-air anomaly from satellite) profile *across* the subduction zone they have computed trench-*parallel* topography and gravity anomalies (TPTA and TPGA, respectively – here renamed to TPaTA and TPaGA to avoid confusion with arc-perpendicular variations). Their findings around the Pacific plate have demonstrated that the largest earthquakes have mostly occurred in areas where both TPaTA and TPaGA are negative. In these areas the plate interface is located relatively deeper, and larger shear tractions accumulate on the down-dip part of the locked zone, resulting in larger stick-slip events^7^. The steeper flexural geometry is reflected in the ocean bottom bathymetry, which has a relatively deeper topography and especially lower gravity signal, even if sediments accumulate in the topographic low. Song and Simons speculate that the structure of the subduction zone, and therefore of TPaTA and TPaGA, should be stable over a million-year time scale.

As subduction velocity is larger than continental convergence rate (by a roughly estimated factor of 4–5 on average), and as the total length of subduction zones is longer than that of collision zones (by a roughly estimated factor of 4–5 as well), large subduction-zone earthquakes occur more frequently: out of 17 M8.5+ earthquakes since 1900 only 1 was in a collisional setting. This means that the two contexts, subduction and collision, are roughly equally active when comparing them by the area of subducted material. As oceanic plate structure is in general simpler (and therefore laterally more homogeneous) than continental, subduction zone earthquakes may reach extremely large magnitudes, for which we have no equivalent instrumentally recorded event in collision zones. However, in terms of seismic risk, collision zones are prone to much larger devastation and damage as population and infrastructure are located literally on and very close to the rupture zone. Therefore assessing seismic segmentation in a continental collision setting, especially in the Himalayas, is essential to evaluate seismic hazard.

In our study, motivated by the approach presented by Song and Simons^7^, we compute arc-parallel topography and gravity anomalies (APaTA and APaGA) along the 2500-km long Himalayan orogen. Song and Simons’ study used free-air anomalies, an appropriate choice as all of the topography is found beneath sea-level, and also because of complete free-air gravity data availability from satellites on sea. For studying a collisional orogen we opt for Bouguer anomaly data, on one hand to eliminate the effect of masses above sea-level to be able to focus on deep structural features, on the other hand because of practical reasons (see sections on Data and Methods). Especially: how the lateral variations of these relate to instrumental and historical seismicity, whether there is any causal link between them, and how the foreland basins reflect these variations?

## Data and New Measurements

### Gravity Data Compilation

Song and Simons^7^ used ETOPO-5 digital elevation model for bathymetry/topography and marine satellite gravity data-derived free-air anomalies^8^ to perform their study. Today, bathymetry as well as marine gravity field derived from satellite altimetry are available at a resolution of ~10 km and an accuracy of ~2 mGal (ref. 9). In a collisional setting high-resolution topography data is readily available from the SRTM mission^10^, but contrary to subduction zones continental satellite gravity data remains to be low-resolution. Therefore the challenge of constituting a comprehensive dataset hinges on gravity data. Satellite gravity datasets from missions such as GRACE and GOCE have a resolution of ca. 100 km (ref. 11), which is similar to the width of the Himalayas (ca. 250 km) and the width of the seismically locked zone (ca. 100 km). Global gravity models such as EGM2008 (refs 12 and 13) claiming higher resolution are created using interpolation, therefore studies based on such models (e.g. ref. 14, with application to the Himalaya) cannot resolve features smaller than 100 km wavelength. We hence aim to build an as complete as possible land gravity dataset to carry out the highest resolution plausible analysis.

**Figure 1:**
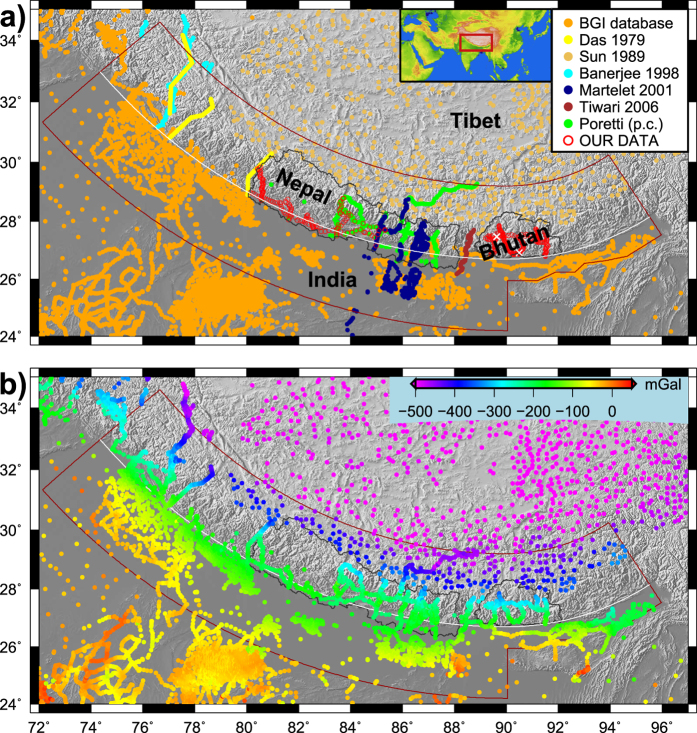
Gravity data. (**a**) Sources of our gravity data compilation (11470 points on map), including new absolute gravity points in Bhutan (white crosses). See main text for description and references. Inset: location of maps in this study. (**b**) Bouguer anomaly map of the Himalayas and surrounding region, referenced to sea-level and using 2670 kg/m^3^ reduction density. Brown contour is the limit of our study area. White arc approximates the topographic front (mostly the Main Frontal Thrust). Boundaries of Nepal and Bhutan are shown in black as reference. Map created with GMT software^39^ version 4 (http://gmt.soest.hawaii.edu/).

We start from our previous compilation of gravity data^15^, based on data from the International Gravimetric Bureau (BGI, http://bgi.omp.obs-mip.fr/), and refs 16, 17, 18. This dataset has a good coverage on either side of the Himalayas, but only a few profiles across the orogen. Therefore we carried out targeted field campaigns to complete this dataset with new field measurements in Nepal^19^ and Bhutan^20^. Finally, we include gravity anomaly data from further three sources. Data by ref. 21 were digitized from projected profiles of the publication. Data reported in ref. 22 were kindly made available by the authors. Unpublished data by G. Poretti was also made available for our study (personal communication). The distribution of the source of gravity data is shown in Fig. 1a.

The consistency of the eight different datasets was verified by producing a series of profiles across the orogen. The relative gravity anomaly dataset of ref. 21 was shifted by −210 mGal to fit the earlier compilation (see details in ref. 15), and two out of five profiles were discarded as found inconsistent with neighbouring datasets. The gravity data from ref. 22 and Poretti (pers. comm.) have been reprocessed in the same manner as our own new data in Bhutan and Nepal. Finally, our hitherto relative gravity anomaly dataset in Bhutan^20^ was calibrated by three absolute gravity bases (see description below) and fully reprocessed using the GravProcess software^23^, resulting in a ca. −20 mGal shift (−19.9 ± 1.7 (1σ) mGal) compared to ref. 20.

### Absolute Gravity Measurement in Bhutan

In March 2015, we have established the first three absolute gravity measurements in Bhutan, two in Thimphu and one in Gelephu, a small city in south central Bhutan (Table 1, Fig. 1a). We have used a Micro-g LaCoste FG5 absolute gravimeter^24^, which has an accuracy of 2 μGal.

To ensure high-quality absolute measurements, gravity has been recorded during 24 hours, with one set of measurement every 30 minutes, and 100 drops per sets at the two stations in Thimphu. In Gelephu the series have been terminated after 7.5 hours of recording due to a too elevated ambient temperature; this should not affect our measurements as the two measurements in Thimphu have demonstrated that tidal effects are very well corrected for by the selected synthetic model. During the calculation of absolute gravity, the recorded data have been corrected for polar motion, pressure variations, Earth tides (ETGTAB^25^) and ocean load (FES2004^26^).

The FG5 instrument provides an absolute gravity value at about 120 cm height above ground. We transfer this to the benchmark on the floor by estimating the vertical gravity gradient in a series of relative gravity (Scintrex CG5 instrument) measurement loops between the floor and 120 cm height^27^. A similar protocol has been used to connect the absolute gravity value with the relative gravity network: gravity ties have been done between the absolute benchmarks and the reference stations of the relative gravity surveys^23^.

**Figure 2:**
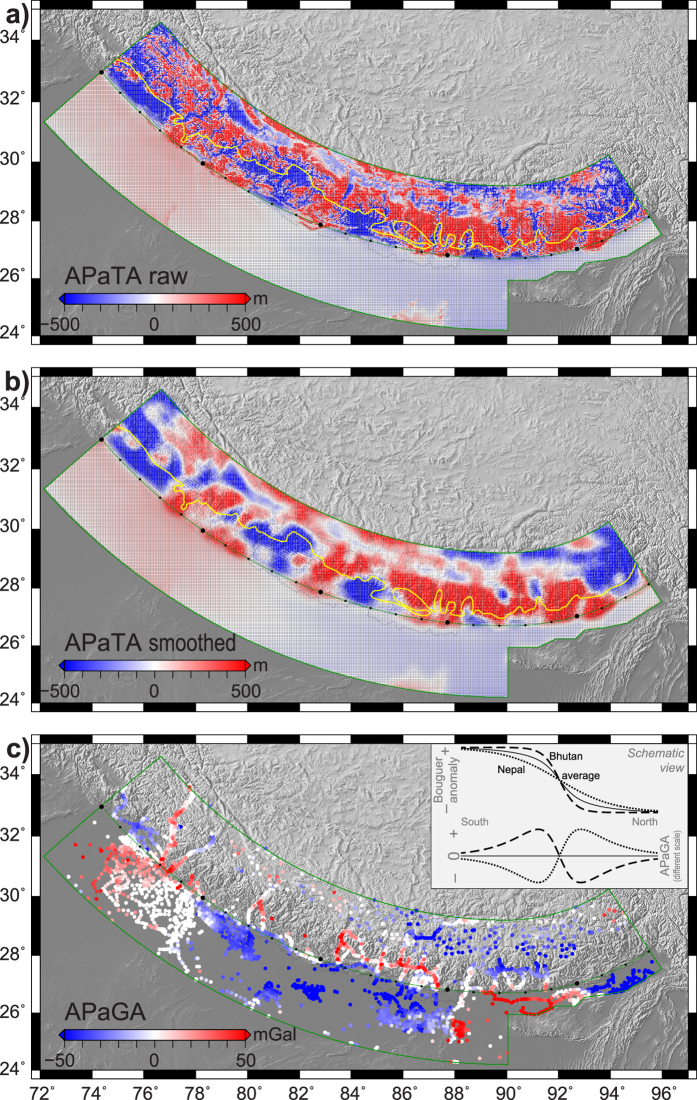
Arc-Parallel Topography and Gravity Anomalies (APaTA and APaGA). Red and blue values represent respectively higher and lower values of topography and gravity compared to the average profile perpendicular to the Himalayan arc. (**a**) APaTA in its raw format. Yellow line marks the Main Central Thrust, the boundary between the Lesser and Higher Himalaya formations (digitised from ref. 28). (**b**) APaTA after smoothing with a 30-km radius circle. (**c**) APaGa in the study area. Inset schematically shows how APaGA is determined: it is the residual anomaly (dashed and dotted lines are examples) compared to the cross-orogen gravity anomaly profile averaged along the orogen (solid line). Black dots on arc approximating the topographic front are placed every 100 km (every 5^th^ larger) as reference for Fig. 3. Map created with GMT software^39^ version 4 (http://gmt.soest.hawaii.edu/).

Our final new gravity dataset in Nepal and Bhutan is available from the authors and will be available through BGI (http://bgi.omp.obs-mip.fr/). In summary, we constructed an unprecedented gravity dataset in the Himalayan region (Fig. 1a), which is presented in terms of Bouguer anomalies (Fig. 1b). The gravity as well as topography data are processed as described in the Methods section to obtain the respective arc-parallel anomalies (Fig. 2).

## Results

### Arc-Parallel Topography Anomaly

The variation of raw APaTA exceeds 1000 m north of the topographic front, in the high range of the Himalayas. Lower values (not saturated colours) are only present north of the range itself, mostly in the central part of the arc. Along the southern part of the Himalaya there are a number of alternating negative and positive patches in the West, while in the East APaTA is mostly positive with a number of narrow negative features.

While these narrow negative APaTA features correspond to deeply eroded valleys in Eastern Nepal, Sikkim and Bhutan, the larger patches can be explained with arc-normal variations. It is simply local relief between the Lesser Himalaya and the Higher Himalaya formations. The Main Central Thrust, which marks the boundary between two formations (Fig. 2a; ref. 28) correlates visually well with this change of APaTA polarity. The sedimentary basin in NW India extending further north than our central arc explains the large negative patch there.

The smoothed APaTA map (Fig. 2b) clearly shows the same features: the topographic relief and the re-entrant in NW India are clearly visible. However, the narrow valleys are filtered out.

South of the topographic front the APaTA values are roughly an order of magnitude smaller (100 m range). Beyond a few topographic features clearly present, there is an overall trend in the Ganges Basin going from high values in the West to low values in the East, showing the long-wavelength topographic dip of the sedimentary basin.

### Arc-Parallel Gravity Anomaly

The variation of APaGA exceeds 100 mGal. There are distinct along-arc segments when one looks at the variation of gravity from the foreland into the orogen:

- In northwesternmost India APaGA is positive in the foreland and negative in the orogen;
- There is a change around 77°E, gradual in the foreland, the sharpness in the orogen is not well constrained;
- All of Nepal is the opposite: negative-to-positive from foreland to orogen;
- There is a change around ca. 88°E, sharp in the foreland and little more gradual in the orogen;
- Bhutan is again positive-to-negative from foreland to orogen;
- There is a change around 93°E, visible in the foreland (there is no data within the Himalayas, but there is also a change further north near 94°E);
- APaGA is negative in the Assam foreland, with a few positive data points north of the Himalayas.

**Figure 3:**
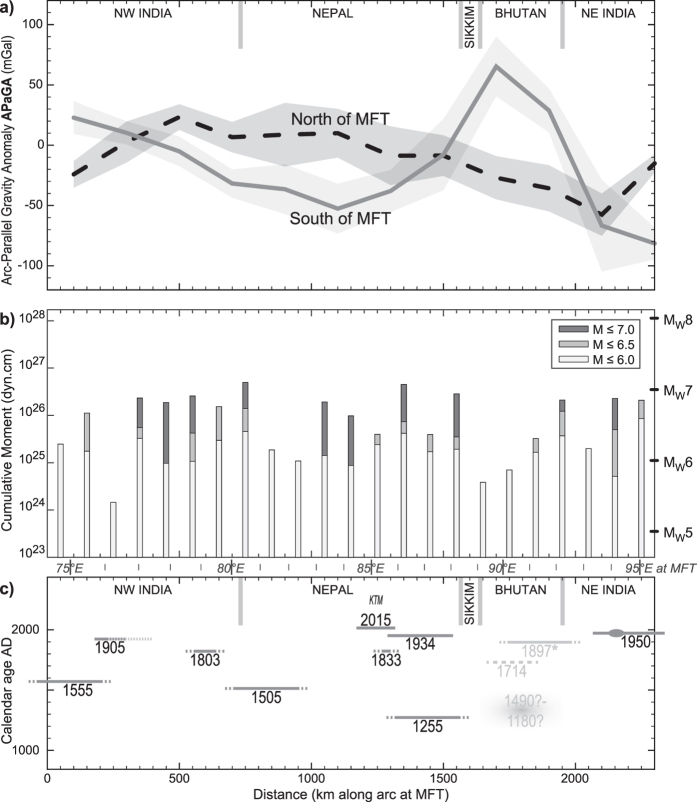
Variations of gravity and seismicity along the Himalayan arc. Horizontal distance is measured along the arc approximation of the topographic front within the study area (see maps on other figures); reciprocally, longitudes at the arc approximating the topographic front are indicated between panels (b,c) as reference. (**a**) Arc-Parallel Gravity Anomaly (APaGA) on either side of the topographic front. Lines represent the averages to at least 25 km to the South, and between 25 and 145 km to the North; shaded areas represent the 1σ deviation of values within each 200-km long bin. Approximate country boundaries are indicated as reference. (**b**) Amount of energy released by moderate to strong (but neither major nor great) earthquakes. Cumulative moment is in dyn.cm, equivalent moment magnitude is shown on right axis. (**c**) Known major and great earthquakes in the Himalaya. Base figure is from ref. 29. Star notes that the 1897 earthquake occurred outside of the Himalaya, at the Shillong Plateau. The 1714 earthquake location is speculative. The medieval event in Bhutan is after refs 3 and 31. The 1905 Kangra earthquake rupture extent is from ref. 40, dotted line is reported from ref. 29. The epicentre was on the western end of the segment and rupture propagated eastwards (see Fig. 4). The knot on the 1950 Assam earthquake represents the 1947 M7.3 event. Other instrumentally recorded events on this diagram are the 1905 M7.9 Kangra earthquake, the 1934 M8.0 Bihar earthquakes, and the 2015 M7.8 and M7.3 events in the Kathmandu (KTM) region. Magnitudes as of the USGS catalogue (http://earthquake.usgs.gov/earthquakes/search/).

At the current level of data coverage, the foreland-to-orogen variation of APaGA is negative-to-positive at the area of Nepal and NE India, and is positive-to-negative near Bhutan and NW India. This is also represented on Fig. 3a as an anti-correlation of APaGA values south and north of the topographic front along the arc. Despite some spread in the gravity data in Nepal due to merging various datasets, the APaGA averages with 1σ deviation are distinct on either side of the orogen front. The two curves cross each other at ca. 350, ca. 1500 and ca. 2100 km along-arc distance (respective longitudes at the topographic front: ca. 77°E, 88°E and 93°E, with ca. ±1° when considering the 1σ zones).

### Seismicity Data

To compare our results with Himalayan seismicity in the discussion (see next section) we show the pattern of both instrumental and historical events along the orogen (Fig. 3b,c).

Figure 3b shows the energy (cumulative moment) released by instrumentally recorded earthquakes. We take the USGS catalogue as events smaller than M4 that are detected by local networks do not contribute to this picture significantly. We aim to choose the maximum magnitude for this graph so that the along-arc comparison makes sense, i.e. the catalogue is complete for events of that magnitude. Irrespective of taking M6.0 (likely complete), M6.5 or M7.0 (likely incomplete) as higher bound, the pattern is the same: there are clear lows at ca. 250, ca. 900 and ca. 1700 km along-arc distance (resp. 76°E near Kangra, 81–83°E in West Nepal and 90°E in Bhutan). With the equivalent Mw-scale on the right it is clear that these zones have not only never produced an instrumental M6 event, but also that the cumulated effect of all smaller events does not reach that of a single M6 event. At these zones either (1) aseismic slip accommodates more of the convergence than in other parts of the orogen, or (2) more strain has accumulated than elsewhere, or (3) the instrumental record is not representative of the long-term seismic cycle.

Figure 3b complements this picture by showing the major and great earthquakes along the Himalayan arc, following ref. 29: three events (2015, 1950, 1934) with relatively well-defined rupture zones, and earlier events with more uncertain bounds. Note that at the longitude of Bhutan (1) the 1897 event occurred at the Shillong Plateau and not in the Himalayas; (2) the 1714 is speculative both in magnitude and location^30^; and (3) the 1180–1490 earthquake has been documented in two paleoseismological sites in south central Bhutan only^3^,^31^.

## Discussion

### Comparison To Subduction Zones

While the respective ranges of arc-parallel topography and gravity anomalies in the Himalayas are the same as in oceanic subduction zones (few 100 to 1000 m, few 10 to 100 mGal), they are far from being correlated (Fig. S1). Therefore zones where both anomalies are negative cannot be used to discuss the location of large earthquakes in the Himalayas. Major events also do not clearly correlate with the low values of either of the anomalies: large earthquakes are known all along the Himalayas, and areas with relatively smaller events (Western Nepal, Bhutan) do show neither the same APaTA nor APaGA pattern. We therefore conclude that Song and Simons’ approach is not directly applicable to continental collision.

The explanation lies, on one hand, in the nature of APaTA: it is primarily controlled by erosion (locally incised valleys) and the geological structure of the Himalayas (relief between Lesser and Higher Himalaya formations). Submarine erosion is slower and spatially more homogeneous than subaerial, resulting in different topography signals for an oceanic (simple) and an orogenic (complex) prism. On the other hand, the intrinsic relationship between bathymetry and free-air gravity on sea is not present in our study, as with Bouguer anomalies we have removed the effect of masses above sea-level, on purpose, to be able to focus on the deep structure of the orogen.

### Interpretation

The arc-parallel gravity anomalies presented here do not correlate with instrumental seismicity (Fig. 3a,b). The low seismic moment release zone at ca. 250 km distance has near-zero APaGA both north and south of the topographic front. The second zone at ca. 900 km has low/high APaGA south/north of the topographic front. The third zone at ca. 1700 km is the opposite.

However, there is a correlation between APaGA and large earthquakes (Fig. 3a,c). Locations where there is a flip of APaGA polarity north and south of the topographic front (Fig. 3a, ca. 350–1500–2100 km, resp. ca. 77–87–93°E) mark out boundaries across which major seismic events do not rupture laterally. The eastern segment boundary sees the 1950 Assam earthquake rupture stopping east of it, with no or uncertain rupture zones in Bhutan. The middle segment boundary near Sikkim also sees the 1255 earthquake rupture zone stop west of it. Finally, the western segment boundary in NW India is near the 1905 Kangra earthquake: this event has initiated at the western end of its rupture area, clearly west of this boundary, and rupture propagated eastward until or into this boundary zone (discussion of this continues below).

**Figure 4:**
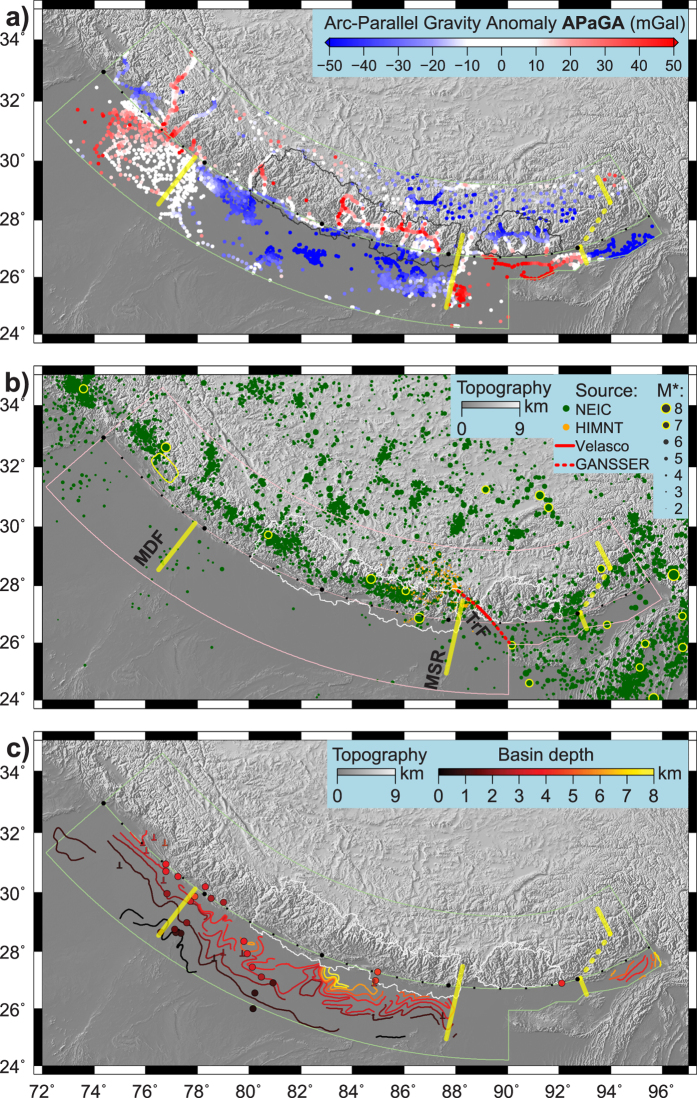
Spatial comparison of APaGA, seismicity and foreland basin depth. Study area and central arc as in Fig. 1, black dots on arc approximating the topographic front are placed every 100 km (every 5^th^ larger) as a reference for Fig. 3. (**a**) Arc-Parallel Gravity Anomaly (from Fig. 2c). Red and blue values represent respectively higher and lower values of gravity compared to the average profile perpendicular to the Himalayan arc. Yellow lines highlight along-arc changes in APaGA, which are then reported on the two subsequent figures. (**b**) Distribution of seismicity. Colour code refers to the source of the data: NEIC is the ANSS Comprehensive Catalogue maintained by the USGS (http://earthquake.usgs.gov/earthquakes/search/), HIMNT project data ref. 41. TrF is the transfer (or transform) fault zone as suggested by seismicity based on ref. 35 and GANSSER project data^36^. Star indicates that magnitude scales are not homogeneous across the three catalogues. Earthquakes with magnitude larger than 7 are highlighted with yellow contour. The 1905 Kangra earthquake rupture contour^40^ is shown in yellow. MDF: Mahendragar-Dehradun Fault. MSR: Munger-Saharsa Ridge. (**c**) Foreland basin depth. Isopach contours are from ref. 32. Inverted T symbols denote borehole data^42^,^43^,^44^,^45^. Circles are estimates from receiver functions^46^,^47^,^48^,^49^. Map created with GMT software^39^ version 4 (http://gmt.soest.hawaii.edu/).

APaGA variations suggest a primary structural segmentation at depth. The four segments defined by flipping foreland-to-orogen APaGA described above (Figs 2c and 3a) clearly point to different flexural geometry and behaviour of the underthrusting India plate. Homogeneity of the India plate flexure within Nepal was already demonstrated by ref. 31 using thermo-mechanical modelling, the same as the difference between Nepal (long flexural wavelength) and Bhutan (short flexural wavelength) by ref. 20. Here we spatially constraint the location of the transition, which is possible thanks to the use of land gravity data. Furthermore, we here document two further segments of the Himalayas by APaGA variations, to obtain a total of four (Fig. 4a): NE India, Bhutan, Nepal (plus India until Dehradun), NW India. From the APaGA pattern we infer that Nepal and NE India start to bend farther south of the topographic front, and disappear beneath the Himalaya at a relatively lower angle, while NW India and especially Bhutan begin to bend closer to the topographic front (farther north) and dip at a steeper angle. Segment boundaries where APaGA indicate major changes are first drawn in yellow on Fig. 4a, and then reported to Fig. 4b,c.

Lateral variation in structure is also reflected in the depth of the sedimentary basin (Fig. 4c). The Ganges Basin south of Nepal is broad and deep, in line with a longer flexural wavelength. The Brahmaputra Basin south of Bhutan is narrow and shallow, flexure occurs on a shorter wavelength. Note that APaGA values south of the topographic front cannot be the effect of the sedimentary basin only, as in NW India there are high APaGa values above a ca. 4 km deep basin.

We therefore conclude that APaGA primarily reflects the lateral variations in the deep structure of the India plate and of the Himalayan collision zone. The western boundary near 77°E, aligned with the Mahendragar-Dehradun Fault^32^ (also referred to as Delhi-Haridwar Ridge), is also visible in the topography. The middle boundary near 88°E, aligned with the eastern end of the Munger-Saharsa Ridge^32^, is also visible in the topography and is the clear termination of the deep foreland basin. This is certainly a major boundary in (or of) the India plate. Whether it has any relationship to the Yadong-Gulu Rift cannot be established here, APaGA values do not vary across the rift, and the Yadong Cross Structure^33^ has not been documented to reach deep in the crust. The eastern boundary near 93°E, east of Bhutan in Arunachal Pradesh, connects to the dextral Kopili Fracture Zone in the foreland (e.g. ref. 34).

The continuation of these boundaries into the orogen in map view (Fig. 4a) is clear for the middle one (at 88°E) and speculative for the two others (although certainly present, see Fig. 3a). The gravity data coverage in NW India does not allow a clear statement, moreover the topographic re-entrant and the presence of Siwalik sediments west of 76°E causes low APaGA values. In NE India (Arunachal Pradesh) there is also a lack of data, however one can could connect the boundary in the foreland with that in the orogen (dotted line on Fig. 4).

The argument to do so is supported by the pattern of seismicity: east of Bhutan, in Arunachal Pradesh, there is a clear, seismically active band that extends across the foreland into the orogen. On the western end of Bhutan, a similar but much more localized, seismically active line was suggested^35^ in the SW corner of the Kingdom, and recently confirmed on a much longer extent^36^. This transfer (and potentially transform) fault zone extends from the north of Sikkim to the northwestern corner of the Shillong Plateau^36^. Thus the Shillong block appears to be cut from both the India plate and the Arunachal Pradesh block, and may behave separately in terms of deformation and flexure. GPS observations also point towards such micro-blocks in NE India^37^. Their boundaries, at depth and at surface, will need to be defined with denser geophysical field campaigns in the future, and block rotations made compatible with the observed pattern and sense of seismicity. New gravity points in Arunachal Pradesh and around the surface trace of the aforementioned transfer fault zone will refine our APaGA analysis in these areas.

While the boundary between the India plate and the Shillong block appears to strike NW-SE, the APaGA pattern reveals a sharp structural boundary within the India plate at 88°E in the N-S direction. The zone East of this area, roughly triangular in shape starting in Sikkim and between ca. 88°E and 90°E in the foreland, produces a few earthquakes (more than the foreland of Nepal), however little can be said about its nature from available data. Since the extents of large earthquakes’ rupture areas in and south of Bhutan are not well constrained (Fig. 3c), we cannot define this segment boundary of the Himalayas more precisely. This segment boundary cuts the presumed rupture zone of the eventual M9 event in 1100 AD suggested by paleoseismology. However, neither this super-earthquake nor its inability to rupture through a segment boundary have been hitherto documented.

Finally, the western segment boundary in NW India also produces a few small earthquakes in the foreland (Fig. 4b). The 1905 Kangra earthquake respects this boundary as the full rupture area is located west of it (Fig. 4b). Instrumental seismicity is misleading in the sense that the “gap” of lower activity is located where the 1905 Kangra event has ruptured, which is further west than the segment boundary. It seems that the instrumental seismicity pattern is not representative of the lateral segmentation of the orogen. Note, however, the cluster of seismicity extending into the Himalayan orogen, turning northwards as a continuation of the Mahendragar-Dehradun Fault.

Figures 3c and 4b highlight that known M7+ earthquakes in the Himalayas occur away from the segment boundaries identified with APaGA. This implies that the structurally different segments also put limits on the lateral propagation of rupture during megathrust earthquakes. From our results two segment lengths can be quantified: the Nepal block is over 1000 km at the topographic front, the Bhutan block is 250–500 km long (depending on the block boundaries being at the transfer fault zone or at 88°E in the west, and on what is selected in the seismicity cluster in the east). While large co-seismic slips on relatively shorter segments can still produce large magnitude events (e.g. ref. 38), we consider that the long-term seismic hazard in Nepal is higher than in Bhutan, especially that part of the accumulated strain may be released at the Shillong Plateau, and not in Bhutan itself, as presumably during the 12 June 1897 M8.3 (USGS) earthquake.

## Conclusions

We have compiled the to-date most comprehensive field-based gravity anomaly database for the Himalayan orogen and investigated arc-parallel topography and gravity anomalies (APaTA and APaGA). While APaTA mainly reflect relief and erosional effects within the Himalayas, APaGA reflect the deep structure of the orogen and its clear lateral segmentation. Four blocks are outlined: NE India, Bhutan, Nepal & India until Dehradun, and NW India. The segment boundaries in the India plate are related to inherited structure (Mahendragar-Dehradun Fault, Munger-Saharsa Ridge), while in the Eastern Himalayas the Shillong block boundaries are highlighted by focused seismic activity. The segmentation of the orogen into these blocks is also reflected in the mean features of the sedimentary foreland basin. We found that the cumulated seismic moment of instrumental seismicity does not highlight the boundaries found by APaGA, likely because the observation time is not representative of the full seismic cycle. However, large earthquakes of the past millennium do not propagate across the segment boundaries defined by lower plate inherited structure. Therefore the here identified segments are not only disparate in terms of flexural behaviour, but also seem to set an upper limit for the lateral extent of potential megathrust earthquakes. Further geophysical and paleoseismological measurements are needed to constrain the rupture extent of past events along the Himalayas, and to better characterize the nature of transition between the here defined segments.

## Methods

We define our study along the Himalayan orogen. As the curvature of the mountain belt is different west and east of longitude 90°E, we approximate the arc with two small circles centred at latitudes 42°N and 35°N respectively, joining each other at longitude 90°E (Fig. 1). The central arc approximating the topographic front of the Himalayas (mostly coincident with the Main Frontal Thrust) is drawn with radii of 15.3° and 8.3° respectively, connecting at latitude 26.7°N at 90°E. On either side of this line a band of 2.5° width is considered^7^, the low-lying India plate to the South, and the deformed orogen to the North. In the South we omit the area of the Shillong Plateau with much higher topography, which cannot be considered when estimating the average topographic profile. The thus defined curved area extends between azimuths 144° and 239° (clockwise from North), spanning from the state of Assam in NE India to NW India, respectively (Fig. 1).

Topography data was taken from the SRTM mission^10^ at 1 arc-minute resolution. This is averaged using 8 km-wide radial bins to compute the mean arc-perpendicular topography profile. For gravity anomaly profile a similar averaging is performed with further binning into 1°-wide azimuthal elements, to avoid artefacts from the uneven distribution of data points. The thus obtained average arc-perpendicular profiles are then subtracted from the original datasets to result in Arc-Parallel Topography and Gravity Anomalies, APaTA and APaGa (Fig. 2a,c). APaTA is also presented in a smoothed version with spatial averaging within a 30 km radius circle (Fig. 2b) to filter out short wavelength variations related to fluvial and glacial erosion.

## Additional Information

**How to cite this article**: Hetényi, G. *et al*. Segmentation of the Himalayas as revealed by arc-parallel gravity anomalies. *Sci. Rep.* **6**, 33866; doi: 10.1038/srep33866 (2016).

## Supplementary Material

Supplementary Information

## Figures and Tables

**Table 1:** Absolute gravity data at the three benchmark stations established in Bhutan.

Station code	AGTDGM	AGTLS	AGGDGM
Location	Thimphu, DGM	Thimphu, LS	Gelephu, DGM
Longitude (°E)	89.635	89.630	90.504
Latitude (°N)	27.474	27.481	26.924
Altitude (m a.s.l.)	2350	2409	284
Vertical gradient (mGal/m)	−0.2639 ± 0.0048	−0.2668 ± 0.0051	−0.2544 ± 0.003
Gravity at the benchmark (μGal)	978′367′276.63	978′357′349.94	978′837′282.28
Date of measurement	11-12.03.2015	13-14.03.2015	16.03.2015

DGM: Department of Geology and Mines. LS: Land Survey.
