# Supplementary material for: Segmentation of the Himalayas as revealed by arc-parallel gravity anomalies
Source: Sci Rep. 2016 Sep 21;6:33866. doi: 10.1038/srep33866 (PMC5030648; doi:10.1038/srep33866)
Supplement: Supplementary Information [file srep33866-s1.pdf]

Supplementary Material for the paper

**Segmentation of the Himalayas as revealed by arc-parallel gravity anomalies**

by

György Hetényi[1,+,x,*], Rodolphe Cattin[2,x], Théo Berthet[3], Nicolas Le Moigne[2], Jamyang Chophel[4], Sarah Lechmann[5,+], Paul Hammer[6,+], Dowchu Drukpa[4], Soma Nath Sapkota[7], Stéphanie Gautier[2], Kinzang Thinley[8]

AFFILIATIONS

1 Institute of Earth Sciences, University of Lausanne, Géopolis, Quartier UNIL-Mouline, 1015 Lausanne, Switzerland

2 Géosciences Montpellier, CNRS UMR5243, Université de Montpellier, Place E. Bataillon, 34095 Montpellier, France

3 Department of Earth Sciences, Uppsala University, Villavägen 16, 75236 Uppsala, Sweden

4 Department of Geology and Mines, Ministry of Economic Affairs, PO Box 173 Thimphu, Bhutan

5 armasuisse, Science & Technology, Federal Department of Defence, Civil Protection and Sport, Feuerwerkerstrasse 39, 3602 Thun, Switzerland

6 SwissRe, Mythenquai 50/60, 8022 Zürich, Switzerland

7 Department of Mines and Geology, Lainchaur, Kathmandu, Nepal

8 National Land Commission, Kawang Jagsa, PO Box 142 Thimphu, Bhutan

+ Formerly at Department of Earth Sciences, ETH Zürich, Soneggstrasse 5, 8092 Zürich, Switzerland

x Formerly at Laboratoire de Géologie, Ecole Normale Supérieure, CNRS - UMR 8538, 24 rue Lhomond, 75005 Paris, France

* Corresponding author: gyorgy.hetenyi@unil.ch

---

**Figure S1.** Arc-Parallel Topography and Gravity Anomalies as a function of profile location along the orogen, north and south of the topography front (TF, closely following the Main Frontal Thrust MFT). The point clouds show that there is rarely a linear and positive correlation between APaTA and APaGA, unlike in the subduction zone cases presented by reference 7 of the manuscript.

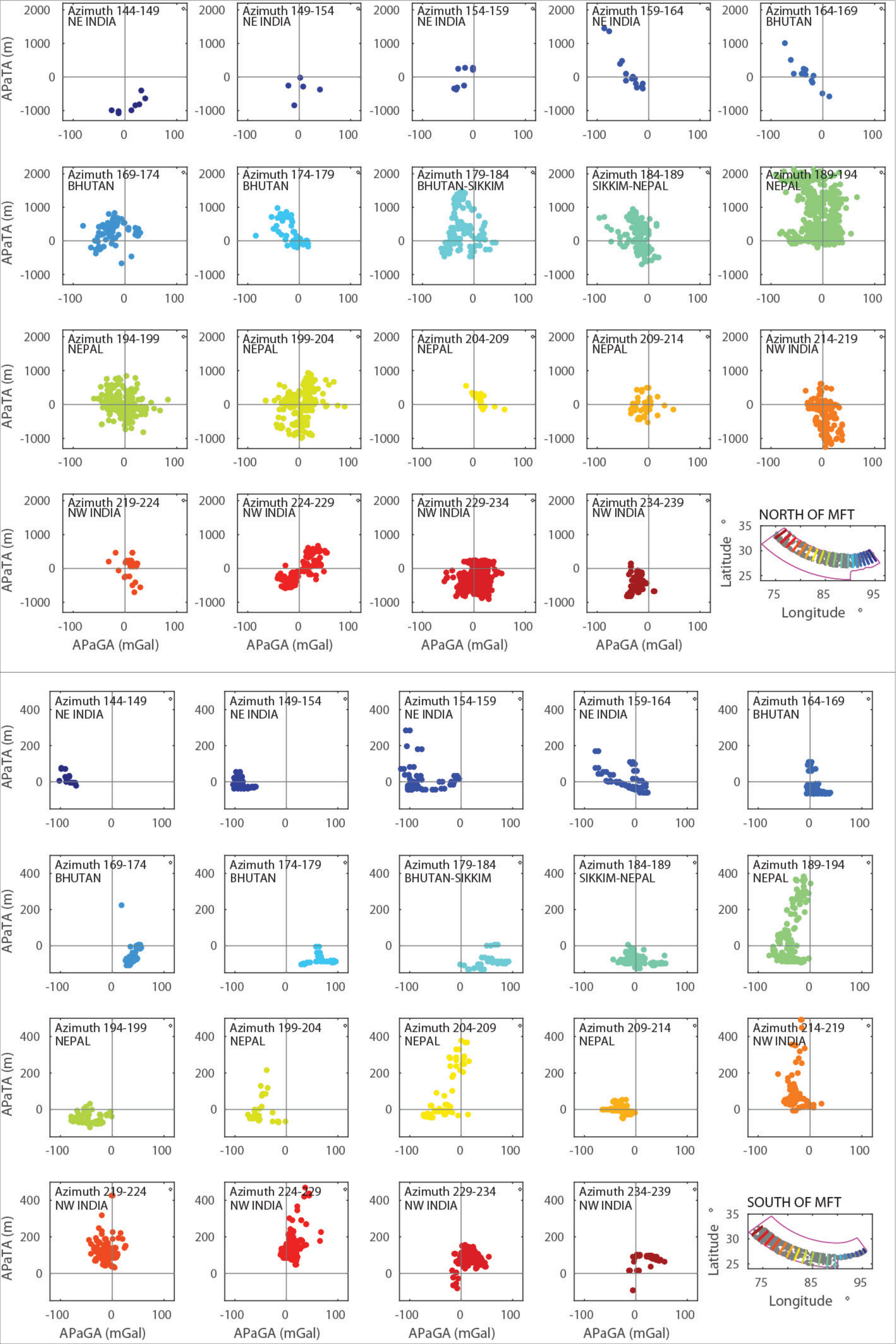

Azimuth 144-149 NE INDIA
Azimuth 149-154 NE INDIA
Azimuth 154-159 NE INDIA
Azimuth 159-164 NE INDIA
Azimuth 164-169 BHUTAN
Azimuth 169-174 BHUTAN
Azimuth 174-179 BHUTAN
Azimuth 179-184 BHUTAN-SIKKIM
Azimuth 184-189 SIKKIM-NEPAL
Azimuth 189-194 NEPAL
Azimuth 194-199 NEPAL
Azimuth 199-204 NEPAL
Azimuth 204-209 NEPAL
Azimuth 209-214 NEPAL
Azimuth 214-219 NW INDIA
Azimuth 219-224 NW INDIA
Azimuth 224-229 NW INDIA
Azimuth 229-234 NW INDIA
Azimuth 234-239 NW INDIA
APaTA (m)
APaGA (mGal)
NORTH OF MFT
SOUTH OF MFT
Latitude °
Longitude °
